# Human Impact on Atolls Leads to Coral Loss and Community Homogenisation: A Modeling Study

**DOI:** 10.1371/journal.pone.0036921

**Published:** 2012-06-05

**Authors:** Bernhard M. Riegl, Charles R. C. Sheppard, Sam J. Purkis

**Affiliations:** 1 National Coral Reef Institute, Nova Southeastern University, Dania Beach, Florida, United States of America; 2 Biology Department, University of Warwick, Coventry, United Kingdom; King Abdullah University of Science and Technology, Saudi Arabia

## Abstract

We explore impacts on pristine atolls subjected to anthropogenic near-field (human habitation) and far-field (climate and environmental change) pressure. Using literature data of human impacts on reefs, we parameterize forecast models to evaluate trajectories in coral cover under impact scenarios that primarily act via recruitment and increased mortality of larger corals. From surveys across the Chagos, we investigate the regeneration dynamics of coral populations distant from human habitation after natural disturbances. Using a size-based mathematical model based on a time-series of coral community and population data from 1999–2006, we provide hind- and forecast data for coral population dynamics within lagoons and on ocean-facing reefs verified against monitoring from 1979–2009. Environmental data (currents, temperatures) were used for calibration. The coral community was simplified into growth typologies: branching and encrusting, arboresent and massive corals. Community patterns observed in the field were influenced by bleaching-related mortality, most notably in 1998. Survival had been highest in deep lagoonal settings, which suggests a refuge. Recruitment levels were higher in lagoons than on ocean-facing reefs. When adding stress by direct human pressure, climate and environmental change as increased disturbance frequency and modified recruitment and mortality levels (due to eutrophication, overfishing, pollution, heat, acidification, etc), models suggest steep declines in coral populations and loss of community diversification among habitats. We found it likely that degradation of lagoonal coral populations would impact regeneration potential of all coral populations, also on ocean-facing reefs, thus decreasing reef resilience on the entire atoll.

## Introduction

Climate change, point-source pollution, increased runoff and overfishing are among the many factors threatening the future of coral reefs. Global estimates put ∼70% of the world’s reefs at various levels of threat and an inexorable rise of human population density near coral reefs [1] suggests that climate change aside, human overuse and pollution are probably among the greatest threats. In general, a greater distance to human habitation often translates into a better state of coral reefs [2], [3], [4], [5]. This has been shown for the NW Hawaiian islands [6], Kiribati [7], [8] Samoa [9], the Chagos [10], New Caledonia [11] and also in the Caribbean [12], [13], although some notable exceptions also exist [14].

For the special case of atoll environments, it has been demonstrated that human habitation can have significantly deleterious effects on their ecological integrity [5],[7],[8],[11] and one might therefore surmise that uninhabitated atolls would lend themselves best for conservation and could potentially serve as refuges for healthy coral reefs [15]. Exactly why this should be so, and what processes would be important, thus merits closer inspection.

Humans interact with reefs in a multitude of ways. At low densities, impacts of human habitation are mainly of local extractive kind, depressing some exploited resource populations but mostly without major impact on the overall system. At high densities, however, significant impacts can be caused by pollution, nutrient enrichment, engineering alterations (dredging for vessel access, filling for housing development, mining for sand and aggregates, etc.), and often-rampant over-exploitation of resources, both living and inanimate [5], [11], [16], [17]. Distant human populations exert far-field pressures via global climate and environmental change that interfere with a multitude of life-processes even on reefs that seem completely removed from direct human contact [5], [18], [45].

The present paper uses a size-based model of coral population dynamics simplified into three functional types determined by growth form and tuned to atolls remote from human habitation (Chagos). It investigates how anthropogenic pressures could potentially alter or harm coral populations. It uses information from the literature to parameterize potential disturbance scenarios and then tracks their consequences. Impacts considered are pollution, nutrification, overfishing and increased siltation/turbidity stress that are expressed via decreased coral recruitment and/or survival rates [7], [11]. In separate models, added to these locally-acting, near-field, stresses are the far-field stressors of global and environmental change that also influence recruitment and cause bleaching mortality [10], [18]. The models are ground-truthed against three decades of monitoring data in the uninhabited Chagos archipelago, where direct, near-field, human impacts are largely absent. Coral population trajectories are first hindcast and then run forward to obtain population parameters as observed during ground-truth (size distributions in 2006, overall cover in 2009). From thereon, population trajectories are forecast under various disturbance scenarios. The model, parameterized to fit dynamics of uninhabited atolls, allows identification of coral demographic processes most at risk and most likely to produce problematic changes if adverse human impacts alone or in concert with climate and environmental change were to act.

## Materials and Methods

### Ethics Statement

This study involved neither direct participation or manipulation of human or animal subjects. It was found to adhere to the Declaration of Helsinki and was approved by the human and animal welfare ethics committees at Nova Southeastern University and the University of Warwick.

### Atoll Study Sites

**Figure 1:**
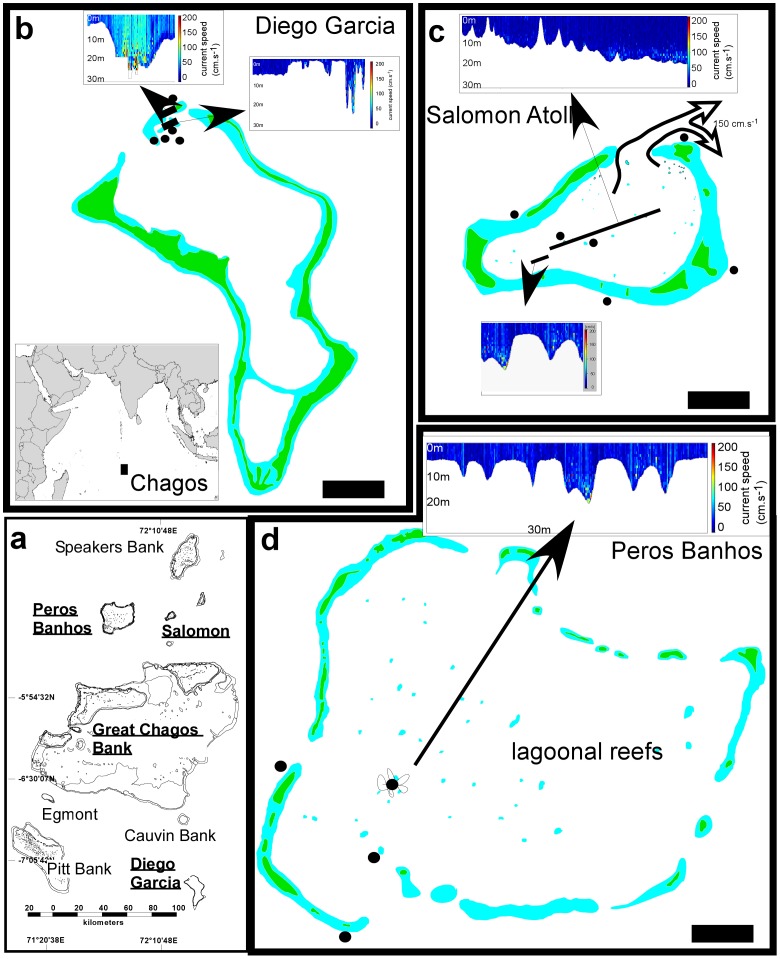
Location of the Chagos archipelago (a) and sampling sites in lagoons an on ocean-facing reefs (b–d). ADCP current measurements are shown as small insets. Current speed is color-coded (see color bar). Black dots show sampling sites.

Atolls were investigated in the Chagos archipelago at Diego Garcia, Peros Banhos, Salomon, Great Chagos Bank, and Egmont (Fig. 1). Regular samples exist from 1979 to 2010 for coral cover. Additionally, coral size distributions were measured from 2006 through 2010. In all locations, coral communities differentiate between reefs inside the central lagoon and the ocean-facing reefs [10], [19] allowing definition of two a priori groups for analysis.

Monitoring data from all Chagos atolls are based on line transects taken from 1979 to 2009 (C.R.C. Sheppard pers.com.), measuring coral cover and sizes as intercepts on reefs inside lagoons and on ocean-facing reefs. In 2006, phototransects were placed in a stratified random pattern at <5 m, 10 m, 20 m, >25 m. Overlapping photographs along a measuring tape created 0.5×20 m photo-corridors. Images were merged, coral outlines digitized, and transferred into binary (black/white) images gridded to unit pixel-size (1 pixel = 1 cm^2^) for each taxonomic category. To allow maximum simplicity and universality of the model, species were lumped into functional types characterized by growth form [20]: massives (slow-growing; all poritids except *Porites cylindrica*, all faviids, fungiids and other massive genera), branching and encrusting forms (fast-growers; most *Acropora*, *Pocillopora*, *Stylophora*, and encrusting or thin-plating forms such as *Montipora, Echinopora lamellosa*) and open arborescent forms (very fast growing; predominantly *Acropora*. In the following only referred to as “arborescent”). For each such growth form, the areas of all individual digitized colonies were evaluated.

Five size classes were used for the models that corresponded to five coral life-stages, defined by radius: spat (<1 cm), recruits (<5 cm), juveniles (<10 cm), corals having reached sexual maturity above a puberty size ( = medium sized, 10–20 cm) and large colonies (>20 cm, open-ended. This size class included some very large colonies). These size-classes are justified by life-history traits [21], [22], [23], [24], [25], [26]. The span of areas for each size class was calculated and measurements from phototransects were binned accordingly. Large corals truncated at edges of the transect image were included if they measured >40 cm diameter at any point or had areas equivalent to 20 cm radius, which automatically included them in the biggest modeled size class. Otherwise, most large corals would have had to be excluded, leading to bias against the large size-class. Small truncated corals were ignored if the shape included in the phototransect did not allow unequivocal estimation of size (i.e. if only a small section of an obviously medium coral fell outside the image and the included area fell within the size-bin, it was still included as a medium coral) since otherwise inclusion might have artificially bolstered smaller size-classes than the truncated corals actually belonged to. Overall, <5% of all encountered corals in size-classes 2–3 were truncated and it is therefore unlikely that significant bias was introduced.

### Population Model

Size-distributions measured in the phototransects were used in a size-based model. The matrix model of coral dynamics [27], [28] could be investigated with and without local recruitment. It was also possible to assume a completely open population [29] since uncertainty exists about local recruitment in coral reefs [30]. The model took the general form:
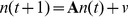
(1)Where *n*(t+1) is a solution vector resulting from multiplication of a size-class transition matrix **A** (containing survival and fertility information of five coral size classes) by a vector of size-distributions at time *n*(t). This latter vector can represent any measured or predicted size-distribution. Added is a vector of zeros (ν) with a single value in position ν_1,1_ that represents recruitment from outside the local population. This value can be held constant, be varied according to specific connectivity assumptions, or randomized. Depending on inclusion of fertility (i.e. positive values) at **A**_1,4_ and **A**_1,5_ (assuming that only size-classes 4 and 5 reproduce) only local (i.e. only from within the sampled population if ν is all zeroes), local and imported (positive values at **A**_1,4 and 5_ as well as at ν_1,1_), or exclusively imported recruitment (**A**_1,4 and 5_ = 0, but a positive value in ν_1,1_) could be modeled. Five size classes were used (see above) and absence of growth by tissue loss due to predation, disturbance, or fragmentation by breakage were considered via transition values in the diagonal [27], [28], [31]. Asexual reproduction could be considered by transition into size class 1 and was thus lumped with sexual recruitment. Fertility was considered to exponentially increase from size class 4 to 5 [31]. This assumption was relaxed in open-population assumptions, where progeny cannot be assigned to a specific size-class.

To be generally applicable to any atoll or other reef system, transition matrices **A** were obtained by sampling existing life-history data and stage transition rules from the Indian Ocean and SE Pacific. Life tables of corals with known and comparable life-history were re-sampled from literature data for calculation of new transition rules using Monte Carlo uncertainty analysis [28], [32]. Survivorship data from the literature and relevant to the five size-classes were combined randomly and independently in 10.000 trials to calculate derived transition rates [28]. Assuming fixed stage durations, following [28] we calculated γ_i_ (probability of growth among stages) from the probability σ_i_, (survival within each stage given in the literature) as:
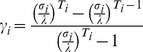
(2)


from which can be obtained the elements of matrix **A** (eq.1) as survival (growth) rates *G* and loops *P* (remaining in the same stage) as
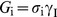
(3)

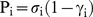
(4)


**Figure 2:**
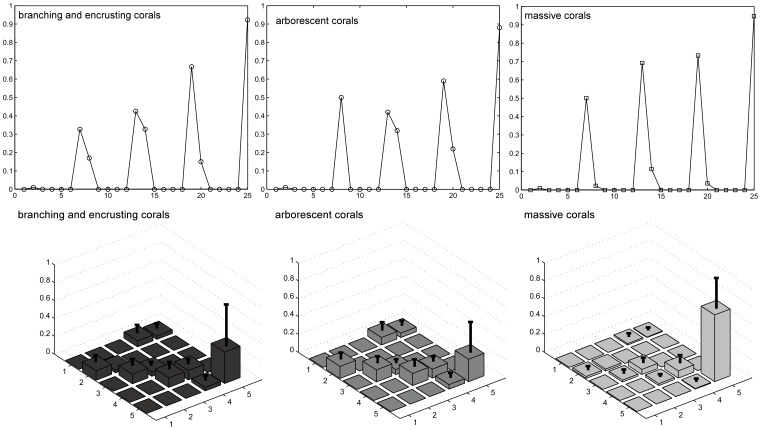
Mean matrices A (upper row) and corresponding elasticity matrices (lower row) for all three growth forms calculated from 10000 Monte-Carlo trials sampling model life tables in five stages. Upper row: The 25 entries of the 5×5 matrix are arranged in column-order. Transitions of settled recruits (A_1,1_…A_5,1_) first, then small corals (A_1,2_…A_5,2_) and so on. A new size class begins at each multiple of 5 (recruits are at positions 1–5, corals <5 cm at positions 6–10, corals 5–10 cm at positions 10–15, etc). Lower row: Elasticity matrices based on transition matrix including variable recruitment. Expressed as means and standard deviations of all elasticity values obtained by substituting all possible combinations of (1, 10, 100, 1000, 10000) into **A**_1,4_ and **A**_1,5_.

Vector Ti contains assumptions of stage durations: for arborescent: [1], [1], [1,2,10]; branching and encrusting: [1], [2], [2], [4,20]; massive [1], [3], [3], [6,24]. Values refer to years spent in each stage. Data for massives were taken from [21], [22], [23]. Arborescent, branching and encrusting from [22], [23], [24], [25], [26]. From each of the 10.000 resampled datasets, the asymptotic growth rate λ was calculated as the dominant eigenvalue. In this type of analysis, spread of λ is an indication of uncertainty and variability [28]. Spread of λ was tight and values always <1, since no fertilities were included. A mean matrix (arithmetic mean, since samples unrelated and from repeat estimation) from all permutations was calculated for each growth form type to be used as the generalized stage-transition model for this paper (Fig. 2).

Model outputs were considered as number of colonies per size class, or as total cover. As a rough indicator for coral cover, colony numbers in each size class were multiplied by the area of a coral in the median of the size class (i.e. radii of 1, 3, 5, 10 cm, assuming a circular area) and then summed.

### Elasticity and Sensitivity Analysis

Elasticity of eigenvalues to changes in stage-transition matrices **A** was calculated following [28] both with and without fertility values at **A**_1,4_ and **A**_1,5_. Minimum and maximum fertility assumptions were derived from field data in all possible combinations and mean elasticity matrices were calculated over all solutions (Fig. 2). Overall model sensitivity to variations in matrix **A** but also to starting population assumptions of survival after the 1998 mass-mortality ( = vector *n*) and levels of connectivity ( = values at ν_1,1_) were evaluated as total proportional deviation of model outcome (i.e. runs from 1999 to 2006 for size distribution, or from 1999 to 2009 for cover data) from the control dataset (2006 size-distributions, 2009 cover data).

### Model Evaluation

The derivation of the matrix model by Monte-Carlo uncertainty analysis of literature data made evaluation of its relevance to field data necessary. Therefore, trajectories from the matrix model were checked against those observed in monitoring. Anchor point for this hindcasting was the mass mortality in 1998. Starting there, it was attempted to obtain the same size distribution and cover of corals by model as observed in the field in 2006. Several coral population trajectories after 1998 were modeled by varying fertility assumptions, and values in population vectors *n* and connectivity vectors ν. Fertilities (**A**_1,4_, **A**_1,5_) and connectivity (ν_1,1_) were randomly and in every possible combination chosen from plausible values (0, 10, 100, 1000, 10000). Predictive success was judged by relative deviation of model- from field-observed size distributions and total frequency. It was attempted to obtain <10% deviation in any size class. Chi-square tests were used. If significant coincidence of modeled and observed data was achieved, it was assumed that population dynamic processes had been adequately modeled.

### Known Impacts

No direct anthropogenic impacts on reefs other than sporadic poaching are reported from the Chagos outside Diego Garcia. However, heavy bleaching mortality in the past is reported [10]. In 1998, most corals bleached on all reef types to ∼30 m depth with near-total mortality in many areas. Lagoonal reefs had more survival than ocean-facing reefs, but it was patchy. Survival in depths >30 m was higher than in shallow water. Virtually the entire shallow *Acropora*-dominated zone was devastated but individual large massive corals survived. In 2002, 2005 and 2010 mild to moderate bleaching without major mortality was observed ([10], Sheppard pers. comm.). Potential anthropogenic and climate change impacts on lagoons were evaluated from the literature and introduced as scaling functions (fractional multipliers to reduce recruitment or survival) into the population models (table 1).

**Table 1:** Human impacts in coral reef lagoons.

Activity	Impacting agent	Action	Effect on corals	Reported reef setting	Source citation	Use in model
Boating	Fuel	Hyrocarbon release	Tissue stress and reduced larvae competence	Worldwidesynthesis	[33]	Reduction in settlement rate, increase in mortality rate
Boating	Antifouling paint	Copper release	Reduced larvae competence	New Caledonia lagoon, laboratory	[11], [34]	Reduction in settlement rate
Habitation	Sewage	Nutrification	Increased algalgrowth	Atolls (Kiribati),Great Barrier Reef	[15], [17], [35]	Reduction in settlement rate
Habitation	Sewage	Introduction ofbacteria andviruses	New pathogens,more diseases	Atolls (Kiribati), Florida Keys	[7], [8], [36]	Increased background and/or episodic coral mortality
Habitation	Fishing	Reduction of herbivorous fish	Increased algalgrowth	Theory based onCaribbean reefs	[37]	Reduction in coral settlement
Habitation	Coastal construction, dredging	Increased turbidityand/orsedimentation	Tissue stress in large corals, reduced settlement	Worldwidesynthesis	[17]	Increased shrinkage and mortality rates, reduced settlement
Climate change	Increased SST	Bleaching events	Pulsed, severe mortality	Worldwidesynthesis	[18]	Increased episodic and/or background mortality
Climate change	Ocean acidification	Lowered aragonite saturation	Reduced larval competence	Laboratory	[38]	Reduced settlement rates
Habitation, Climate and environmental change	Physical disturbance, fertilized water column; changed oceanic fronts,	Increased predator outbreaks	Increased frequency and severity of mortality	Mariana Islands, Great Barrier Reef, Red Sea	[39], [40], [41]	Increased episodic mortality

## Results

### Field Results - Cover Patterns in Guilds

**Figure 3:**
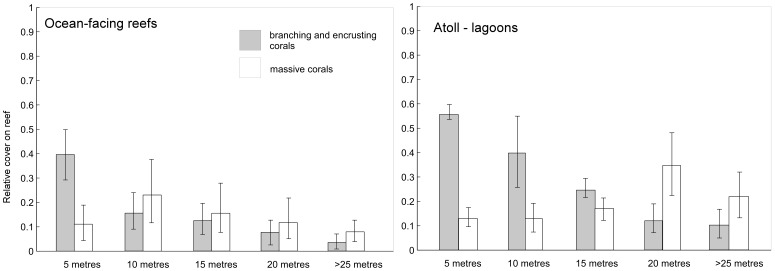
Live coverage of branching and encrusting as well as massive growth forms at sample sites on ocean-facing reefs and in lagoons of the Chagos archipelago. Arborescent forms are included with branching and encrusting forms since they covered significant space mainly in shallow and deep lagoons, but not the other habitats. Error bars are standard deviation.

Branching, encrusting and arborescent corals dominated space from 5 to 10 m on lagoonal reefs, but only at 5 m on ocean-facing and exposed reefs (Fig. 3). *Acropora* dominated the shallow areas <10 m (branching *A. cytherea* and *A. clathrata* on ocean-facing reefs, on lagoonal patch reefs *A. tenuis, A cytherea,* and open arboresecnt *A. formosa*). *Pocillopora* spp. and *Stylophora* spp. were subordinate. In some <10 m localities, encrusting corals (*Montipora* and *Echinopora*) covered as much space as *Acropora*. Large encrusting to whorl-shaped corals were locally found to be common at greater depth (*Echinopora lamellosa* colonies >10 m diameter at ∼30 m depth) in lagoons. Large massives (primarily *Porites* spp.) occurred both at shallow and greater depths, suggesting persistence through the 1998 bleaching event.

### Size Distributions

**Figure 4:**
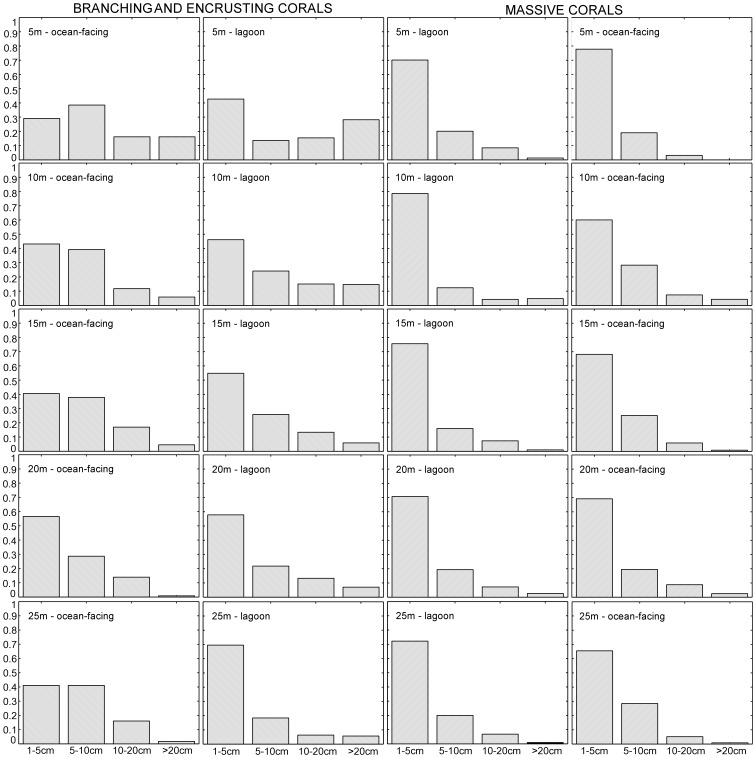
Size distribution of corals in the Chagos. Branching, encrusting and arborescent corals are pooled, since arboresecent corals only occur in specific environments. Values are based on sums of all measured corals in all transects in each environment. The fifth size-class used in models is spat, which cannot be adequately counted in phototransects.

Four size-classes were recorded in phototransects (1–5, 5–10, 10–20,>20 cm diameter) while the fifth and smallest size class, spat and recruits <1 cm, could not be reliably detected. In massive corals, juveniles (1–5 cm) had the highest share of the population at all depths (Fig. 4). Juvenile branching, encrusting and arborescent corals had a lower share in their respective populations on ocean-facing reefs but dominated in the deep lagoons (Fig. 4) while at shallow sites several size classes had similar frequency even with some bimodality. Significantly more small branching and encrusting corals were found on deep lagoon floors (>25 m) than at the same depths on ocean-facing reefs (t-test, p<0.01, Fig. 4). Overall, 4–21/10 m^2^ juveniles of all growth forms were counted on ocean-facing reefs, 0–61/10 m^2^ on lagoonal reefs, demonstrating high variability in both habitat types. Almost twice as many large corals of all growth forms were encountered in lagoons than on ocean-facing reefs, but no clear pattern of increase with depth existed in either setting (R^2^ = 0.16 in lagoons, R^2^ = 0.09 ocean-facing). To correct for juveniles (<5 cm) obscured by larger specimens in phototransects, we also counted juveniles directly in 0.25 m^2^ quadrats. This yielded a mean of 22/m^2^ suggesting a maximum of ∼220 juveniles/10 m^2^ phototransect. Assuming 1% survivorship of spat (Harriott 1985), a settlement of ∼22000 spat per transect would be needed to produce the above value.

### Dynamics of Coral Populations – Unimpacted Case

Using transition matrices for three growth forms (Fig. 2), dynamics leading to observed size distributions were hindcast. The mass mortality of 1998 was used as a starting point from which populations had to recover. Several combinations of survival and recruitment assumptions resulted in modeled size distributions comparable to field measurements (Appendix S1 and S2). Solutions represent the closest fit to field data out of millions of possible outcomes based on variable entry assumptions (levels of survival in 1998, fertility assumptions, local or connected recruitment). These outcomes suggested growth form-specific variability of survival in 1998, of subsequent dynamics, and an overall high connectivity of populations. The recruitment maxima and minima ( = number of spat) required to obtain a fit to field data varied among depths. In 6 of 10 environmental settings, the branching and encrusting coral model provided agreement with field results, in the other environments the arborescent coral model yielded higher predictive accuracy (Appendix S1). Fit of model and field data (measured by Chi-square tests) at shallowest lagoon sites (5 m) could be improved if the largest size class was excluded, suggesting asexual reproduction generating large branching and encrusting corals more rapidly than model predictions. It is also possible the growth rates were under-estimated, however, in that case poorer fit across all size classes would be expected. Also for massive corals, model solutions were satisfactory for all size-classes except the largest (Appendix S2). In this case, the presence of more large corals than could be modeled by assuming zero or very low survival in 1998 clearly suggested survival of some bigger massives (e.g. *Porites*). This was also reported by [10]. It is unlikely that growth rates were underestimated or model results mis-interpreted.

Hindcast model solutions for all growth forms showed poorest fit to field data in deep lagoons (>25 m). High coral cover at this depth where coral growth is slow, can best be explained by absence of severe mortality in 1998 (verified by [10]) suggesting a refuge.

**Figure 5:**
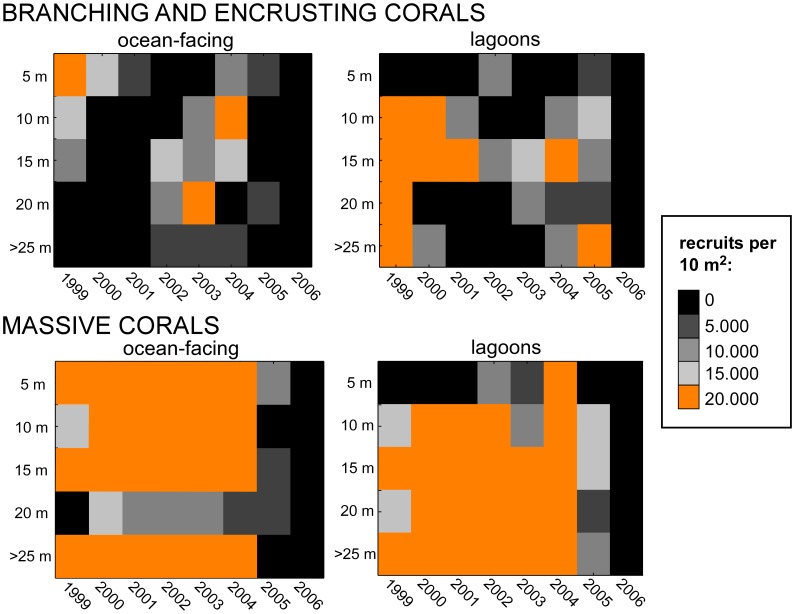
Summary of annual recruitment level ( = number of spat) between 1998 and 2006 required to obtain size distributions as observed in 2006, based on model runs. Matrices show color-coded modal values of number of spat for each year (x-axis) and each depth zone (y-axis). Highest recruitment levels are orange, lowest black. Overall, higher values of recruitment (more orange cells) were predicted for inside the lagoons.

Models assuming mass mortality in 1998 and a combination of local and irregular imported recruitment (0–20000) had ∼50% lower fit to field data than assumption of all recruitment imported from outside the local population. This suggested larval connectivity with another (or several) population(s) being of critical importance. Overall, the model suggested more recruitment (number of spat) in lagoons than on ocean-facing reefs (Fig. 5).

Sensitivity of the overall model to (A) fertility assumptions (values in A_1, 4 and 5_), (B) recruitment assumptions (local versus connected, i.e. zero or positive value in ν_1,1_) and (C) timing of larval import (i.e., in which of the model years between 1998 and 2006 recruitment was held zero or at another value) was evaluated by the relative differences between final output vectors of model runs and field verification data. Sensitivity was (B)>(C)>(A), meaning that variability in the number of externally produced/imported spat resulted in a greater deviation from field data than variation in timing and whether the local population was allowed to recruit into itself or not. This clearly underlines the importance of connectivity.

Comparison of model and field data suggested the following dynamics on Chagos reefs with virtually no human interference:

The mass mortality of 1998 was preferentially survived by large massives.
A refuge with lower mortality existed in deep lagoons.
regeneration occurred via connectivity rather than local recruitment.
Irregular recruitment was sufficient to generate the observed recovery.
Dynamics differed between lagoons and ocean-facing reefs and between different depths.
Highest recruitment was found inside the lagoons.

The assumptions of these dynamics were checked by comparison against a time-series of coral cover on ocean-facing reefs from 1978 to 2009. The long-term dataset had a maximum ∼20% variability around means and suggested an increase in coral cover by ∼10% from 2006–9. The modeled increase in coral cover corresponded to field observations and verified the realism of the model results (Appendix S3).

### Dynamics of Coral Populations – Impacted Case

**Figure 6:**
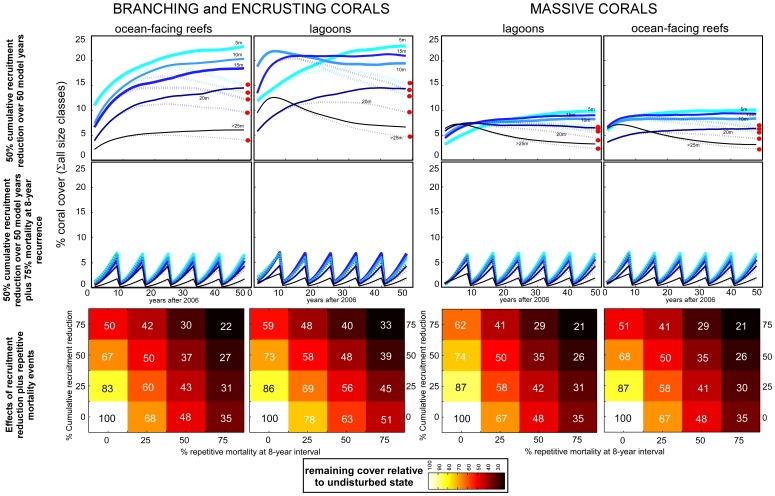
Predictive model outcomes of impacts on coral cover on atolls. Four columns represent lagoonal and ocean-facing coral populations of branching and encrusting and massive corals. Models use recruitment values of Fig. 5, and size distributions of Fig. 3 multiplied by matrices of Fig. 2. This is the general model that was verified by hindcasting dynamics between 1998 and 2006. First row: trajectory of coral cover under annual recruitment reduction of one percent ( = 50% cumulatively over 50 model years). Thick/dotted lines = scenario without/with recruitment reductions, red dots = final degraded coral cover value after 50 model years. Second row: trajectory of coral cover with/without (thick/dotted lines) recruitment reduction (1% annually cumulative over 50 years) and 75% mortality in non-recruit size classes every eight years. Third row: Synergistic effects of recruitment reduction and repeat mortality of non-recruit size classes (2–5) on coral cover. Color coded value is percent of cover remaining relative to completely undisturbed scenario.

Human use of atolls has been shown to impact coral population dynamics by pollution, eutrophication and increased turbidity/sedimentation, disadvantaging coral growth and survivorship and also reducing recruitment (table 1). Overfishing creates ecological cascades disadvantaging settlement via increased algal growth. Additionally, far-field effects of climate change, like bleaching events and disease outbreaks, need to be accounted for. Tabulation of some of the most obvious impacts shows that their net result tends to be a reduction of recruitment and/or increased mortality of larger corals (table 1). Thus, model projections concentrated on these mechanisms and examined effects of each alone, and acting together. Fig. 6 shows projected trajectories over 50 years. In the “better-case-scenario” it was assumed that low levels of water-borne pollutants, some overfishing and increased algal turfs only disadvantage recruitment but did not increase mortality of size-classes 2–4. Recruitment reduction was cumulative and increased annually so that after 50 years either 0, 25, 50 or 75% recruitment was lost. In this scenario, depending on severity, coral cover declined by up to one half in 50 years (Fig. 6 upper row, for scenario with 50% recruitment reduction). In a “worse-case-scenario”, it was assumed that near-field human impacts combined with far-field climate and environmental change effects. Repeat pollution, disease or predator outbreaks and bleaching events would kill a variable amount of corals (0, 25, 50 or 75% at each event) at eight year intervals, a realistic scenario [42], [43], and additionally, that ocean acidification, heat and waterborne pollutants in combination with increased algal growth also equally reduce recruitment. In this case, coral cover would decline drastically (Fig. 6). In model runs with <50% repeat mortality, corals maintained >25% of their original cover with a generally increasing trend over all events. From 50% mortality during each event onwards, populations recovered, but never enough to maintain upward cover trends. Coral cover declined to about 20% of undisturbed cover. Massive corals, due to higher recruitment values (Fig. 5; Appendix S1 and S2), would for a while be repeatedly able to attain comparable cover values as without mass mortality. These populations would be composed of many small, rather than fewer large colonies as in the absence of repeated mortality. However, since recruitment would be reduced every year, the long-term trend would be one of inexorable decline. In the undisturbed and “better-case” scenario, coral cover and space cover by specific growth forms differed markedly between lagoons and ocean-facing reefs (Fig. 6, upper row). Recurrent heavy mortality would cause a homogenization of coral cover (as low in lagoons as on ocean-facing reefs), lowering coral cover and altering coral community structure by preferentially disadvantaging branching, encrusting and arborescent corals, such as *Acropora*. The coral community would change to a more homeogeneous, low-cover and simplified assemblage across lagoons and ocean-facing reefs. These trajectories are comparable to field data from Kiribati atolls [5], [7]. If disturbance frequency was increased to <8 years, the assemblage changed further to even lower cover made up by small branching and small massive corals that never attained large sizes (not illustrated since not expected within the next 50 years).

## Discussion

Coral populations in atoll environments, as on any reef in general, are relatively fragile with regards to impacts from climate and environmental change and other, more direct, human impacts [5]. Studies in the coral atolls of the Line Islands and the NW Hawaiian islands have shown that with increased distance to human habitation, coral cover and fish density tend to increase, and negatives, such as for example coral diseases, decline [5], [7], [8], [44]. Potential causative factors are shown in table 1 and were harnessed for a theoretical examination of several “what if” modeling scenarios of what might happen to coral populations under minimal or maximum anthropogenic pressure, depending on intensity of and proximity to human use. This is of relevance for the Chagos. They are presently the world’s largest marine protected area and are also one of the world’s largest remaining wildernesses without any permanent human habitation [45]. There exists, however, strong debate on whether or not resettlement of the atolls should be allowed. And there is the question what damage far-field human impacts through climate and environmental change can cause on even these most remote reef systems. Better information about potential effects of near- and far-field anthropogenic pressures can only help management decision processes in this specific, and indeed any other, reef setting.

The dynamics of Chagos coral populations between 1998 and 2009 showed differences in population structure and life-history between lagoons and ocean-facing reefs. In lagoons, higher levels of recruitment, higher coral cover and also higher persistence through bleaching events were observed as well as estimated from models. The coral biostromes (laterally continuous, thin coral frameworks [46]) in the deep lagoons were identified as having acted as a refuge due to lower mortality and also high recruitment. Also on shallow lagoonal reefs, more large colonies had survived the 1998 bleaching event than on ocean-facing reefs. Currents measured inside lagoons were much weaker than on ocean facing reefs (Fig. 1) suggesting that the combination of deep coral refuge, individual large survivors, and weak currents may lead to a greater availability and longer residence of larvae in the lagoons, thus causing the higher recruitment levels (Fig. 5). Since models suggested that coral populations across the Chagos must be highly connected, it is possible that lagoonal reefs may also serve to seed ocean-facing reefs and might therefore be a locus of resilience for the entire atoll reef system. Of course differences in assemblage structure exist between lagoonal and ocean-facing reefs [19], some coral species may have unique dynamics to either environment and some components of ocean-facing communities may be decoupled from lagoonal processes. However, many of the dominant species on Chagos reefs occur both inside and outside the lagoons [10], [19], suggesting that connectivity may indeed play an important role.

Lagoons may be at more immediate risk from human and climate change impacts than ocean-facing reefs [11], although evidence for the opposite also exists [47]. However, nutrification and pollution due to human settlement affects lagoons particularly strongly and frequently leads to algal proliferation that in turn reduces coral recruitment [11], [7]. Other forms of pollution may also have negative effects. Models suggest that even in the absence of acute coral mortality, cover would be expected to decrease in the long term in response to human pressure if coral recruitment is disadvantaged by as little as 1% per annum. Scenarios exclusively disadvantaging recruitment resulted in coral cover declines of about 30% in 50 years (Fig. 6) due to lacking replacement of losses by natural mortality in big colonies. This scenario not only bears relevance to local land-based impacts but also to climate and environmental change (both heat and acidification reduce larval viability/settlement; [38], [49]). Not surprisingly, if background mortality of corals is raised (for example due to pollution, sedimentation stress, predators or diseases), coral declines are much more dramatic. From the models it is evident that impacts from even a sparse human population have the potential to reduce coral cover.

Introduction of repeated heavy mortality events, such as caused by bleaching, predator or disease outbreaks [18], [42], drastically decreased contribution of branching, encrusting and arborescent species. In the models, massives fared better due to higher recruitment. But massives are also frequently more bleaching-resistant, less susceptible to diseases and less favored by predators [39], [47], [48], [49]. Thus, under repeated heavy mortality, whether caused by climate and environmental change or local human habitation, the coral community will change to a novel low-cover assemblage with changed contribution by growth forms. Due to the decline in coral diversity, differences in communities among environments can be lost (Fig. 6, middle row). The direction of this change will depend on causes, patterns, and frequency of such mass mortality, but the models and experience [47], [49] suggest that community changes are indeed likely to ensue. Even in the absence of any local human population, far-field effects of anthropogenic climate and environmental change can affect recruitment and mortality, and therefore have the potential to irreversibly reduce coral cover and cause changes towards novel, homogenized assemblage structure that lacks previously observed differentiation. Any such changes driven by far-field pressures would be dramatically augmented by the addition of significant near-field stresses.

Model results also find support in studies showing faster coral reef regeneration in mostly unimpacted areas, such as marine reserves, where higher fish and herbivore densities keep settling substrates available for coral recruits [50]. The largely unexploited nature of the Chagos and its good fish populations [51] presents as healthy an environment for coral regeneration as can be found anywhere. Since recovery in the Chagos from the 1998 bleaching event was faster than in most other Indian Ocean reef areas [10], this is a strong argument to support the minimization of human impacts on coral reefs and furthering their protection.

## Supporting Information

Appendix S1 **Results of the hindcasting model 1- Branching/encrusting and arborescent corals, presented for each environment; Data colums 1–5 show modeled frequencies in coral size classes, columns 6–13 show estimated number of spat in each model year.** First and second line shows maxima and minima, third, italicized line is the mode (used for forecast models). Accuracy of model prediction ( = percent range of predicted value deviation from observed values) is shown in third line of text column1. Ocean = Ocean-facing reef; Lagoon = lagoonal reef; Lagoon-5 m could only be solved if size-class 5 was ignored. * = not considered to evaluate model fit.
(DOCX)

Appendix S2 **Results of the hind-casting model 2 - massive corals; presented for each environment; Data colums 1–5 show modeled frequencies in coral size classes, columns 6–13 show estimated number of spat in each model year.** First and second line shows maxima and minima, third, italicized line is the mode (used for forecast models). Accuracy of model prediction ( = percent range of predicted value deviation from observed values) is shown in third line of text column1. Ocean = Ocean-facing reef; Lagoon = lagoonal reef. * = not considered to evaluate model fit.
(DOCX)

Appendix S3 **Forecast solutions linking 2006 size-class data and population projections to the 2009 cover data.** The increases in column 7 are comparable to those observed in the field [10]. Values in columns 2, 3, and 4 are those recruitment values from a large number of trials that led to the best-fitting increase assumption. In two cases, several equally acceptable solutions existed. The growth form-dependent model (arborescent, branching and encrusting, or massive corals) is identified in column one.
(DOCX)
