# Supplementary material for: Human Impact on Atolls Leads to Coral Loss and Community Homogenisation: A Modeling Study
Source: PLoS One. 2012 Jun 5;7(6):e36921. doi: 10.1371/journal.pone.0036921 (PMC3367966; doi:10.1371/journal.pone.0036921)
Supplement: Appendix S1 — Results of the hindcasting model 1- Branching/encrusting and arborescent corals, presented for each environment; Data colums 1–5 show modeled frequencies in coral size classes, columns 6–13 show estimated number of spat in each model year. First and second line shows maxima and minima, third, italicized line is the mode (used for forecast models). Accuracy of model prediction ( = percent range of predicted value deviation from observed values) is shown in third line of text column1. Ocean = Ocean-facing reef; Lagoon = lagoonal reef; Lagoon-5 m could only be solved if size-class 5 was ignored. * = not considered to evaluate model fit. (DOCX) [file pone.0036921.s001.docx]

**Appendix S1:**

| **Branching/ encrusting**  **arborescent** | **Size 1** | **Size 2** | **Size 3** | **Size 4** | **Size 5** | **1999** | **2000** | **2001** | **2002** | **2003** | **2004** | **2005** | **2006** |
| --- | --- | --- | --- | --- | --- | --- | --- | --- | --- | --- | --- | --- | --- |
| **Ocean-5m**  arborescent  20% | 0 | 50 | 47 | 19 | 19 | 15000 | 0 | 0 | 0 | 0 | 5000 | 5000 | 0 |
|  | 20000 | 50 | 67 | 29 | 29 | 20000 | 20000 | 20000 | 15000 | 10000 | 10000 | 5000 | 20000 |
|  | ***0*** | ***50*** | ***57*** | ***29*** | ***19*** | ***20000*** | ***15000*** | ***5000*** | ***0*** | ***0*** | ***10000*** | ***5000*** | ***0*** |
|  |  |  |  |  |  |  |  |  |  |  |  |  |  |
| **Ocean-10m**  branching  15% | 0 | 66 | 48 | 15 | 7 | 0 | 0 | 0 | 0 | 0 | 15000 | 0 | 0 |
|  | 20000 | 79 | 54 | 22 | 10 | 20000 | 20000 | 20000 | 20000 | 20000 | 20000 | 0 | 20000 |
|  | ***0*** | ***78*** | ***48*** | ***22*** | ***7*** | ***15000*** | ***0*** | ***0*** | ***0*** | ***10000*** | ***20000*** | ***0*** | ***0*** |
|  |  |  |  |  |  |  |  |  |  |  |  |  |  |
| **Ocean-15m**  branching  10% | 0 | 59 | 52 | 24 | 6 | 0 | 0 | 0 | 0 | 5000 | 10000 | 0 | 0 |
|  | 20000 | 68 | 55 | 29 | 8 | 15000 | 20000 | 20000 | 20000 | 20000 | 20000 | 0 | 20000 |
|  | ***0*** | ***68*** | ***52*** | ***29*** | ***7*** | ***10000*** | ***0*** | ***0*** | ***15000*** | ***10000*** | ***15000*** | ***0*** | ***0*** |
|  |  |  |  |  |  |  |  |  |  |  |  |  |  |
| **Ocean-20m**  branching  10% | 0 | 75 | 33 | 19 | 1 | 0 | 0 | 0 | 10000 | 20000 | 0 | 5000 | 0 |
|  | 20000 | 75 | 33 | 19 | 1 | 0 | 0 | 0 | 10000 | 20000 | 0 | 5000 | 20000 |
|  | ***0*** | ***75*** | ***33*** | ***19*** | ***1*** | ***0*** | ***0*** | ***0*** | ***10000*** | ***20000*** | ***0*** | ***5000*** | ***0*** |
|  |  |  |  |  |  |  |  |  |  |  |  |  |  |
| **Ocean>25m**  branching  15% | 0 | 24 | 20 | 10 | 1 | 0 | 0 | 0 | 5000 | 5000 | 5000 | 0 | 0 |
|  | 20000 | 26 | 22 | 10 | 1 | 0 | 0 | 5000 | 10000 | 5000 | 5000 | 0 | 20000 |
|  | ***0*** | ***24*** | ***20*** | ***10*** | ***1*** | ***0*** | ***0*** | ***0*** | ***5000*** | ***5000*** | ***5000*** | ***0*** | ***0*** |
|  |  |  |  |  |  |  |  |  |  |  |  |  |  |
| **Lagoon-5m**  arborescent  20% | 0 | 50 | 14 | 16 | *2 | 0 | 0 | 15.000 | 0 | 0 | 0 | 5000 | 0 |
|  | 20000 | 50 | 14 | 61 | *51 | 20000 | 20000 | 20000 | 15000 | 0 | 0 | 5000 | 20000 |
|  | ***0*** | ***50*** | ***17*** | ***37*** | ***24*** | ***0*** | ***0*** | ***0*** | ***10000*** | ***0*** | ***0*** | ***5000*** | ***0*** |
|  |  |  |  |  |  |  |  |  |  |  |  |  |  |
| **Lagoon-10m**  arborescent  20% | 0 | 150 | 55 | 34 | 34 | 5000 | 0 | 0 | 0 | 0 | 5000 | 15000 | 0 |
|  | 20000 | 150 | 83 | 52 | 46 | 20000 | 20000 | 20000 | 20000 | 15000 | 15000 | 15000 | 20000 |
|  | ***0*** | ***150*** | ***63*** | ***52*** | ***35*** | ***20000*** | ***20000*** | ***10000*** | ***0*** | ***0*** | ***10000*** | ***15000*** | ***0*** |
|  |  |  |  |  |  |  |  |  |  |  |  |  |  |
| **Lagoon-15m**  branching 15% | 0 | 189 | 71 | 48 | 16 | 20000 | 15000 | 5000 | 0 | 15000 | 20000 | 10000 | 0 |
|  | 20000 | 194 | 75 | 49 | 17 | 20000 | 20000 | 20000 | 20000 | 20000 | 20000 | 10000 | 20000 |
|  | ***0*** | ***189*** | ***72*** | ***48*** | ***16*** | ***20000*** | ***20000*** | ***20000*** | ***10000*** | ***15000*** | ***20000*** | ***10000*** | ***0*** |
|  |  |  |  |  |  |  |  |  |  |  |  |  |  |
| **Lagoon-20m**  branching 10% | 0 | 78 | 25 | 19 | 8 | 10000 | 0 | 0 | 0 | 10000 | 5000 | 5000 | 0 |
|  | 20000 | 79 | 26 | 19 | 8 | 20000 | 15000 | 5000 | 5000 | 10000 | 5000 | 5000 | 20000 |
|  | ***0*** | ***78*** | ***25*** | ***19*** | ***8*** | ***20000*** | ***0*** | ***0*** | ***0*** | ***10000*** | ***5000*** | ***5000*** | ***0*** |
|  |  |  |  |  |  |  |  |  |  |  |  |  |  |
| **Lagoon>25m**  arborescent  10% | 0 | 200 | 53 | 17 | 15 | 20000 | 0 | 0 | 0 | 0 | 10000 | 20000 | 0 |
|  | 20000 | 200 | 57 | 21 | 17 | 20000 | 20000 | 15000 | 5000 | 0 | 10000 | 20000 | 20000 |
|  | ***0*** | ***200*** | ***54*** | ***19*** | ***15*** | ***20000*** | ***10000*** | ***0*** | ***0*** | ***0*** | ***10000*** | ***20000*** | ***0*** |

Appendix S1: Results of the hindcasting model 1- Branching/encrusting and arborescent corals, presented for each environment; Data colums 1-5 show modeled frequencies in coral size classes, columns 6-13 show estimated number of spat in each model year. First and second line shows maxima and minima, third, italicized line is the mode (used for forecast models). Accuracy of model prediction (=percent range of predicted value deviation from observed values) is shown in third line of text column1. Ocean=Ocean-facing reef; Lagoon=lagoonal reef; Lagoon-5m could only be solved if size-class 5 was ignored. *=not considered to evaluate model fit.
