# Supplementary material for: Human Impact on Atolls Leads to Coral Loss and Community Homogenisation: A Modeling Study
Source: PLoS One. 2012 Jun 5;7(6):e36921. doi: 10.1371/journal.pone.0036921 (PMC3367966; doi:10.1371/journal.pone.0036921)
Supplement: Appendix S2 — Results of the hind-casting model 2 - massive corals; presented for each environment; Data colums 1–5 show modeled frequencies in coral size classes, columns 6–13 show estimated number of spat in each model year. First and second line shows maxima and minima, third, italicized line is the mode (used for forecast models). Accuracy of model prediction ( = percent range of predicted value deviation from observed values) is shown in third line of text column1. Ocean = Ocean-facing reef; Lagoon = lagoonal reef. * = not considered to evaluate model fit. (DOCX) [file pone.0036921.s002.docx]

Appendix S2:

| **Massives** | **Size 1** | **Size 2** | **Size 3** | **Size 4** | **Size 5** | **1999** | **2000** | **2001** | **2002** | **2003** | **2004** | **2005** | **2006** |
| --- | --- | --- | --- | --- | --- | --- | --- | --- | --- | --- | --- | --- | --- |
| **Ocean-5m**  1% | 0 | 249 | 56 | 20 | *2 | 0 | 0 | 5000 | 15000 | 20000 | 20000 | 10000 | 0 |
|  | 20000 | 256 | 62 | 29 | *4 | 20000 | 20000 | 20000 | 20000 | 20000 | 20000 | 10000 | 20000 |
|  | ***0*** | ***252*** | ***56*** | ***25*** | ***3*** | ***20000*** | ***20000*** | ***20000*** | ***20000*** | ***20000*** | ***20000*** | ***10000*** | ***0*** |
|  |  |  |  |  |  |  |  |  |  |  |  |  |  |
| **Ocean-10m**  20% | 0 | 156 | 62 | 29 | *4 | 15000 | 20000 | 20000 | 20000 | 20000 | 20000 | 0 | 0 |
|  | 20000 | 156 | 63 | 30 | *4 | 20000 | 20000 | 20000 | 20000 | 20000 | 20000 | 0 | 20000 |
|  | ***0*** | ***156*** | ***62*** | ***29*** | ***4*** | ***15000*** | ***20000*** | ***20000*** | ***20000*** | ***20000*** | ***20000*** | ***0*** | ***0*** |
|  |  |  |  |  |  |  |  |  |  |  |  |  |  |
| **Ocean-15m**  30% | 0 | 206 | 63 | 30 | *4 | 20000 | 20000 | 20000 | 20000 | 20000 | 20000 | 5000 | 0 |
|  | 20000 | 306 | 63 | 30 | *4 | 20000 | 20000 | 20000 | 20000 | 20000 | 20000 | 20000 | 20000 |
|  | ***0*** | ***206*** | ***63*** | ***30*** | ***4*** | ***20000*** | ***20000*** | ***20000*** | ***20000*** | ***20000*** | ***20000*** | ***5000*** | ***0*** |
|  |  |  |  |  |  |  |  |  |  |  |  |  |  |
| **Ocean-20m**  1% | 0 | 109 | 31 | 5 | *0 | 0 | 0 | 0 | 0 | 5000 | 5000 | 0 | 0 |
|  | 20000 | 111 | 31 | 20 | *3 | 20000 | 20000 | 20000 | 20000 | 20000 | 20000 | 5000 | 20000 |
|  | ***0*** | ***109*** | ***31*** | ***18*** | ***2*** | ***0*** | ***15000*** | ***10000*** | ***10000*** | ***10000*** | ***5000*** | ***5000*** | ***0*** |
|  |  |  |  |  |  |  |  |  |  |  |  |  |  |
| **Ocean>25m**  1% | 0 | 156 | 63 | 30 | *4 | 20000 | 20000 | 20000 | 20000 | 20000 | 20000 | 0 | 0 |
|  | 20000 | 156 | 63 | 30 | *4 | 20000 | 20000 | 20000 | 20000 | 20000 | 20000 | 0 | 20000 |
|  | ***0*** | ***156*** | ***63*** | ***30*** | ***4*** | ***20000*** | ***20000*** | ***20000*** | ***20000*** | ***20000*** | ***20000*** | ***0*** | ***0*** |
|  |  |  |  |  |  |  |  |  |  |  |  |  |  |
| **Lagoon-5m**  1% | 0 | 107 | 31 | 5 | *0 | 0 | 0 | 0 | 0 | 0 | 0 | 0 | 0 |
|  | 20000 | 109 | 31 | 21 | *3 | 20000 | 20000 | 20000 | 20000 | 20000 | 20000 | 5000 | 20000 |
|  | ***0*** | ***107*** | ***31*** | ***11*** | ***2*** | ***0*** | ***0*** | ***0*** | ***10000*** | ***5000*** | ***20000*** | ***0*** | ***0*** |
|  |  |  |  |  |  |  |  |  |  |  |  |  |  |
| **Lagoon-10m**  1% | 0 | 276 | 44 | 15 | *1 | 0 | 0 | 0 | 0 | 10000 | 5000 | 15000 | 0 |
|  | 20000 | 282 | 44 | 28 | *4 | 20000 | 20000 | 20000 | 20000 | 20000 | 20000 | 20000 | 20000 |
|  | ***0*** | ***278*** | ***44*** | ***18*** | ***2*** | ***15000*** | ***20000*** | ***20000*** | ***20000*** | ***10000*** | ***20000*** | ***15000*** | ***0*** |
|  |  |  |  |  |  |  |  |  |  |  |  |  |  |
| **Lagoon-15m**  50% | 0 | 155 | 33 | 5 | *0 | 0 | 0 | 0 | 0 | 0 | 0 | 0 | 0 |
|  | 20000 | 356 | 63 | 30 | *4 | 20000 | 20000 | 20000 | 20000 | 20000 | 20000 | 20000 | 20000 |
|  | ***0*** | ***275*** | ***37*** | ***17*** | ***2*** | ***20000*** | ***20000*** | ***20000*** | ***20000*** | ***20000*** | ***20000*** | ***15000*** | ***0*** |
|  |  |  |  |  |  |  |  |  |  |  |  |  |  |
| **Lagoon-20m**  1% | 0 | 204 | 58 | 22 | *2 | 0 | 5000 | 15000 | 20000 | 20000 | 20000 | 5000 | 0 |
|  | 20000 | 206 | 62 | 29 | *4 | 20000 | 20000 | 20000 | 20000 | 20000 | 20000 | 20000 | 20000 |
|  | ***0*** | ***204*** | ***60*** | ***25*** | ***3*** | ***15000*** | ***20000*** | ***20000*** | ***20000*** | ***20000*** | ***20000*** | ***5000*** | ***0*** |
|  |  |  |  |  |  |  |  |  |  |  |  |  |  |
| **Lagoon>25m**  80% | 0 | 234 | 59 | 24 | *2 | 0 | 10000 | 15000 | 15000 | 15000 | 15000 | 10000 | 0 |
|  | 20000 | 356 | 63 | 30 | *4 | 20000 | 20000 | 20000 | 20000 | 20000 | 20000 | 20000 | 20000 |
|  | ***0*** | ***254*** | ***59*** | ***27*** | ***4*** | ***20000*** | ***20000*** | ***20000*** | ***20000*** | ***20000*** | ***20000*** | ***10000*** | ***0*** |

Appendix S2 : Results of the hind-casting model 2 - massive corals; presented for each environment; Data columns 1-5 show modeled frequencies in coral size classes, columns 6-13 show estimated number of spat in each model year. First and second line shows maxima and minima, third, italicized line is the mode (used for forecast models). Accuracy of model prediction (=percent range of predicted value deviation from observed values) is shown in third line of text column1. Ocean=Ocean-facing reef; Lagoon=lagoonal reef. *=not considered to evaluate model fit.
