# Supplementary material for: Human Impact on Atolls Leads to Coral Loss and Community Homogenisation: A Modeling Study
Source: PLoS One. 2012 Jun 5;7(6):e36921. doi: 10.1371/journal.pone.0036921 (PMC3367966; doi:10.1371/journal.pone.0036921)
Supplement: Appendix S3 — Forecast solutions linking 2006 size-class data and population projections to the 2009 cover data. The increases in column 7 are comparable to those observed in the field [10]. Values in columns 2, 3, and 4 are those recruitment values from a large number of trials that led to the best-fitting increase assumption. In two cases, several equally acceptable solutions existed. The growth form-dependent model (arborescent, branching and encrusting, or massive corals) is identified in column one. (DOCX) [file pone.0036921.s003.docx]

Appendix S3:

|  | **Spat, 2007** | **Spat, 2008** | **Spat, 2009** | **2006 cover** | **2009 cover** | **% increase** |
| --- | --- | --- | --- | --- | --- | --- |
| **arborescent_5m** | 0 | 10000 | 0 | 42 | 40 | -5 |
| **massive_5m** | 15000 | 20000 | 15000 | 16 | 17 | 6 |
|  | 20000 | 0 | 15000 | 17 | 18 | 6 |
| **branching_10m** | 0 | 10000 | 20000 | 19 | 21 | 11 |
| **massive_10m** | 20000 | 0 | 20000 | 24 | 24 | 0 |
| **branching_15m** | 0 | 0 | 20000 | 15 | 16 | 7 |
| **massive_15m** | 10000 | 15000 | 20000 | 18 | 18 | 0 |
| **branching_20m** | 15000 | 20000 | 15000 | 9 | 10 | 11 |
| **massive_20m** | 15000 | 0 | 0 | 14 | 15 | 7 |
|  | 20000 | 15000 | 0 | 14 | 15 | 7 |
|  | 5000 | 5000 | 5000 | 14 | 15 | 7 |
| **branching>25m** | 0 | 10000 | 0 | 3 | 4 | 33 |
| **massive>25m** | 5000 | 5000 | 0 | 10 | 11 | 10 |

Appendix S3: Forecast solutions linking 2006 size-class data and population projections to the 2009 cover data. The increases in column 7 are comparable to those observed in the field [10]. Values in columns 2, 3, and 4 are those recruitment values from a large number of trials that led to the best-fitting increase assumption. In two cases, several equally acceptable solutions existed. The growth form-dependent model (arborescent, branching and encrusting, or massive corals) is identified in column one.
